# Nutrient Content and Compliance with Sodium Standards in Elementary School Meals in the United States Pre- and Post-COVID-19

**DOI:** 10.3390/nu14245386

**Published:** 2022-12-19

**Authors:** Leah Elizabeth Chapman, Scott Richardson, Amanda A. Harb, Evan Fear, Tara P. Daly, Deborah A. Olarte, Meghan Hawley, Emelia Zukowski, Colin Schwartz, Meghan Maroney, Juliana F. W. Cohen

**Affiliations:** 1 Center for Health Inclusion, Research, and Practice (CHIRP), Department of Public Health and Nutrition, Merrimack College, 315 Turnpike Street, North Andover, MA 01845, USA; 2 Department of Nutrition, Harvard T.H. Chan School of Public Health, 677 Huntington Ave, Boston, MA 02115, USA; 3 Department of Health and Behavior Studies, Teachers College, Columbia University, New York, NY 10027, USA; 4 Department of Biochemistry, College of Science, Northeastern University, 360 Huntington Ave, Boston, MA 02115, USA; 5 Center for Science in the Public Interest, Washington, DC 20005, USA

**Keywords:** school meals, nutrition, breakfast, lunch, sodium, COVID-19

## Abstract

Various federal policies have weakened school meal nutrition standards in the United States since the passage of the Healthy, Hunger-Free Kids Act in 2010, including temporary school meal nutrition waivers to promote post-COVID-19 pandemic recovery. This study used school menu and nutrient data from a nationally representative sample of 128 elementary school districts to examine differences in nutrients (average calories, total fat, saturated fat, sodium, total sugar, and fiber) and alignment with United States Department of Agriculture (USDA) sodium targets in 2019 (pre-pandemic) and in 2022 (post-pandemic). Data were analyzed using analysis of variance accounting for repeated measures within school districts, adjusting for geographic region and urbanicity. Small differences in the nutrient content for both breakfast and lunch were observed between 2019 and 2022. Most weeks met USDA sodium Target 1 for breakfast (≥95% of weeks) and Target 1 (≥96% of weeks) and Target 1A for lunch (≥92% of weeks) in both 2019 and 2022, although compliance decreased slightly when condiments were included. Additionally, meals provided on average 57 g of total sugar. Overall, many meals are already in alignment with lower sodium targets. Simple strategies, such as offering lower sodium condiments, can further reduce sodium in school meals. The total sugar levels observed highlight that the USDA should consider limits on added sugars in school meals.

## 1. Introduction

In the United States (U.S.), nearly all public and non-profit private schools participate in the U.S. Department of Agriculture’s (USDA) federal school meal programs, which include the National School Lunch Program (NSLP) and School Breakfast Program (SBP) [1,2]. Prior to the COVID-19 pandemic, roughly 30 million children received school meals daily, with many students consuming up to half of their daily energy intake at school [3,4]. Given the large number of students eating breakfast and/or lunch at school (and the substantial amount of time students spend in schools [approximately 7 h daily]), setting strict nutrition standards for school meals has the potential to make meaningful improvements in the diet quality of school-aged children [5].

In 2010, the Healthy, Hunger-Free Kids Act (HHFKA) strengthened nutrition standards for school meals, including requirements for more whole-grain-rich foods; a variety of vegetables offered during the week; larger portion sizes for fruits and vegetables; limits on calories; and the elimination of trans fats [6]. Additionally, the HHFKA standards included sodium limits, comprising three targets that were to be phased in over a decade [7]. The Target 1 standards for elementary students (implemented during School Year (SY) 2014–2015) were 540 milligrams (mg) at breakfast and 1230 mg at lunch. Target 2 standards had a planned implementation during SY 2017–2018 with maximum sodium levels of 485 mg at breakfast and 935 mg at lunch for elementary students. The final sodium target (Target 3) was set for SY 2022–2023, with maximum levels of 430 mg at breakfast and 640 mg at lunch for grades kindergarten through fifth (K-5). Supplemental Table S1 summarizes each sodium target by age group and meal type. Overall, due to policies such as the HHFKA, research has found that children often have their most nutritious meals of the day at school [8,9,10].

While there is evidence highlighting the nutritional quality of school meals, as well as the acceptability of the healthier meals and benefits for students [8,9,10,11,12,13,14,15,16,17], federal nutrition standards have been weakened over time, both through temporary and permanent policies (a timeline of recent policy changes is summarized in Supplemental Figure S1). For example, in 2018, the USDA rolled back the standards for the whole-grain-rich requirements (from 100% to 50% whole-grain-rich foods offered), delayed the Target 2 sodium standards from SY 2017–2018 to SY 2024–2025, and eliminated the final Target 3 sodium standards [18]. The USDA reported that these modifications were due to concerns from food service personnel regarding the ability to prepare menus that would align with the updated nutrition standards and be acceptable to students [18].

In response to the COVID-19 pandemic, Congress passed the Families First Coronavirus Response Act (FFCRA) in March 2020 [19]. During this time period, schools throughout the U.S. faced multiple challenges, including the logistical and operational challenges of providing meals to children remotely, as well as budget shortfalls, staffing shortages, and supply chain issues [20]. In response to these difficulties, the FFCRA provided meal-pattern waivers (in addition to other waivers) that allowed schools to waive specific components of the SBP and NSLP meal pattern if these hardships were preventing them from meeting the nutrition standards [19]. These waivers were revised and renewed throughout the pandemic; in October 2021, a revised waiver (“Nationwide Waiver to Allow Specific School Meal Pattern Flexibility for School Year 2021–2022”) was implemented through 30 June 2022 [21]. Concurrently, in April 2020, a federal court ruled against the USDA’s 2018 school meal modifications on procedural grounds, restoring the original three sodium reduction targets and whole-grain-rich requirement (as well as reinstating a ban on flavored 1% milk) [22].

Most recently, to support post-pandemic recovery, in February 2022, the USDA issued a transitional, short-term “bridge rule” for school meals (“Transitional Standards for Milk, Whole Grains and Sodium”) [22]. This policy created a new Target 1A standard for lunch (see Supplemental Table S1 for sodium standards) that will go into effect in SY 2023–2024 for lunch (breakfasts will remain at the original Target 1 standards during this time). This policy was implemented in part to ensure schools have sufficient time to develop recipes and procure foods that align with lower sodium targets. USDA has committed to more comprehensive rulemaking to provide certainty to schools beyond SY 2023–2024 early next year.

Currently, little is known about the impact of the post-pandemic recovery (COVID-19 FFCRA) waivers on school meal nutrition quality, such as calories, total fat, saturated fat, sodium (alignment with the current USDA Target 1 standards), total sugar, and fiber. However, this is important to understand, as policy makers are considering continuations of these waivers, but it is unclear what impact they have on nutrients in school meals. Additionally, more information is needed to understand school meal alignment with stronger USDA sodium targets Target 1A (the standard for lunch starting 1 July 2023) and Targets 2 and 3 (while these are no longer in effect, evaluations can help inform policy decisions for longer-term future targets, which the USDA has committed to in future rulemaking). Therefore, the aims of this research were to (1) examine the average nutrient content of school meals (including calories, total fat, saturated fat, sodium, total sugar, and fiber) before and in the presence of the COVID-19 pandemic waivers (‘post-pandemic’) and to (2) determine the alignment of school meals with USDA sodium targets during these two time periods among a nationally representative sample of elementary schools. These specific nutrients were selected because they are regulated by school meal standards (or because they serve as proxies for school meal standards, such as fiber for whole-grain requirements). As a sub-aim, this study also examined differences in nutrient content and sodium target compliance by region and urbanicity, as school food-service operational challenges may vary in different parts of the U.S.

## 2. Materials and Methods

### 2.1. Data Collection Procedures

A comprehensive list of all non-charter, coeducational public elementary schools serving K-5 students participating in the NSLP in the U.S. was obtained through the National Center for Education Statistics (*n* = 12,736 schools). School districts were selected using stratified random sampling by USDA Food and Nutrition Service Region (*n* = 7 regions [Northeast, Mid-Atlantic, Southeast, Midwest, Mountain Plains, Southwest, and West; Supplemental Figure S2]) and total district K-5 enrollment (*n* = 11 strata by size). Districts were excluded if they did not publish their NSLP menus with nutrition information and were replaced with additional randomly selected districts within the same stratum. When possible, a total of two districts per stratum were selected; however, because there were several strata that did not have a district fitting the size criterion in particular regions (as shown in Supplemental Table S2), a total of 128 districts were included in the final sample.

Urbanicity for each district was determined using the USDA’s Economic Research Service Rural-Urban Commuting Area (RUCA) Codes, which classify U.S. census tracts through measures of population density, urbanization, and commuting. Districts were categorized as: (1) urban (RUCA code 1); (2) suburban (RUCA codes 2–3); (3) large rural (RUCA codes 4–6); and (4) rural/small town (RUCA codes 7–10).

### 2.2. Menu Measures

Four weeks of SBP and NSLP menus were collected from the 128 districts in the fall of 2019 (SY 2019–2020, pre-COVID-19) to provide a representative menu cycle, resulting in *n* = 2865 days of breakfasts and lunches collected across the elementary school districts. In the spring of 2022 (SY 2021–2022, post-COVID-19), an additional four weeks of menus for breakfast and lunch were collected from the original school districts. Among those, *n* = 37 districts no longer provided menus with nutrition information online, resulting in a total sample of *n* = 91 school districts with data for SY 2021–2022 (districts with missing nutrient information were evenly distributed both by region and total district K-5 enrollment [see Supplemental Table S3]), resulting in a sample that remained nationally representative across the USDA Food and Nutrition Service Regions and district sizes).

Menus with their nutrients (calories, total fat, saturated fat, sodium, total sugar, and fiber) were downloaded from the school-district websites. Although there are no federal school meal standards for total fat, total sugar, added sugar, or fiber, these nutrients were examined as potential proxies for other school meal standards (for example, fiber may be a proxy for compliance with whole grain, fruit, and vegetable requirements). Additionally, these nutrients were examined because they are associated with chronic-disease risk factors [23]. Research assistants coded menu items to correspond to the USDA-mandated meal components: entrée (including meat/meat alternative and grain/bread); vegetable; fruit; and fluid milk. Condiments were also coded.

### 2.3. Missing Data

Approximately 4% of foods had missing nutrients. When possible, these values were populated by finding the exact product match from another school district within the dataset. An additional strategy to populate missing nutrient information was to conduct internet searches to find a product’s USDA Child Nutrition label. These internet searches were conducted during the corresponding year of the menu item; thus, if foods were reformulated between 2019 and 2022, the correct nutrient data was recorded. The remaining missing values were populated from similar items within the corresponding district’s menu within the same school year.

Foods with implausible nutrient values were excluded from analyses (e.g., >2000 calories; >53 g of total fat; >19 g saturated fat; >65 g of total sugar; and/or >31 g of fiber). Cut points for implausible food values were determined based on daily intake recommendations (e.g., an individual food would likely not be 2000 calories, as this is the standard calorie intake recommendation for an entire day).

### 2.4. Creating Daily and Weekly Nutrient Totals

After accounting for missing data, menu items were then coded to create average daily nutrients by first averaging the nutrient values for each meal component for each school for each day. For example, if a school district served both pizza and a chef salad as entrées for lunch, those nutrient values were averaged to create that day’s average entrée nutrient values for lunch. Daily averages were then estimated by summing the nutrient averages of each component to create the day’s total nutrient values. Of note, daily nutrient values were summed based on the USDA’s requirements for breakfast and lunch (e.g., for breakfasts, daily values consisted of an entrée [meat/meat alternative AND/OR grain/bread], milk, and fruit OR vegetable; for lunch, meals consisted of an entrée [meat/meat alternative AND grain/bread], milk, fruit, AND vegetable). Weekly averages were then estimated by averaging the daily nutrient values over the week (according to the calendar year).

### 2.5. Statistical Analyses

Adjusted average daily nutrients—calories, total fat, saturated fat, sodium, total sugar, and fiber—for breakfast and lunch meals were estimated using least squares mean regression comparing fall 2019 (pre-COVID-19) to spring 2022. Differences between the two time points were examined using analysis of variance accounting for repeated measures within school districts, adjusting for geographic region and urbanicity. The analysis was restricted to *n* = 91 districts for both 2019 and 2022 to capture changes in nutrients more accurately between the two years (e.g., the 37 school districts that were excluded due to missing menu data in 2022 were excluded from 2019 in analyses comparing the two years).

Additionally, the percent of meals that met, and that were close to meeting (within 10%) of the sodium Targets 1, 1A, 2, and 3 were calculated per day and per week (the USDA considers sodium compliance as an average over the week). When examining the sodium targets, the full samples were analyzed (*n* = 128 districts in 2019 and *n* = 91 districts in 2022). In secondary analyses, alignment with the sodium targets was examined with the addition of condiments. Secondary analyses were also conducted to examine differences by region and urbanicity using logistic regression models [24]. All analyses were conducted using StataIC (version 16.0, 2019, StataCorp LLC, College Station, TX, USA). Results were considered statistically significant if the *p*-value was < 0.05.

## 3. Results

A total of 128 school districts were included in the nationally representative sample, including 14 school districts in the Northeast, 20 in the Mid-Atlantic, 22 in the Southeast, 16 in the Midwest, 17 in the Mountain Plains, 20 in the Southwest, and 19 in the West (Supplemental Table S2). A total of 8941 meals (from approximately 94,000 individual meal components) were examined across the school districts that had data from both 2019 (*n* = 5707 meals among 128 districts) and 2022 (*n* = 3234 meals among 91 districts).

The average calories, total fat, saturated fat, sodium, total sugar, and fiber for school districts’ breakfasts and lunches are presented in Table 1, and Supplemental Table S4 compares average nutrient values with USDA nutrient targets for calories, saturated fat, and sodium (there are no USDA nutrient targets for total fat, total sugar, and fiber for reimbursable school meals). For breakfast meals, most nutrients significantly decreased from 2019 to 2022; calories decreased by 18 kilocalories (kcal) (a 4.8% decrease), saturated fat by 0.2 g (g) (a 3.6% decrease), sodium by 37 mg (a 9.3% decrease), total sugar by 1.1 g (a 3.6% decrease), and fiber by 0.2 g (a 5.6% decrease). Total fat did not significantly change for breakfast (7.2 g vs. 7.0 g; *p* = 0.2). When examining school-district lunches, saturated fat significantly increased by 0.2 g (a 3.5% increase) between 2019 and 2022, while sodium and total sugar significantly decreased (by 42 mg [4.8% decrease] and 1.6 g [5.5% decrease], respectively). Calories, total fat, and fiber did not significantly change for lunch.

In secondary analyses examining differences by region (examining data from both 2019 and 2022), the Western region (e.g., Alaska, California, Hawaii, Idaho, Nevada, Oregon, Washington) had on average less calories, total fat, saturated fat, sodium, and sugar in lunch entrées, compared with the other regions (data not shown). On average, the Southeast region (e.g., Alabama, Florida, Georgia, Kentucky, Mississippi, North Carolina, South Carolina, and Tennessee) had the highest levels of sodium (903 mg compared with 757 mg in the Western region; *p* < 0.0001) and the lowest average levels of fiber (whereas the Northeast region [e.g., Connecticut, Maine, Massachusetts, New Hampshire, New York, Rhode Island, Vermont] had the highest average fiber levels; 6.6 g vs. 9.4 g; *p* < 0.0001). The Northeast region also had the highest average levels of total sugar (30.6 g vs. 23.1 g in the Western region; *p* < 0.0001). Regional trends at breakfast were similar to those observed at lunch, with lower overall nutrient values (reflecting that breakfast is a smaller meal with fewer required meal components). However, compared with lunch, similar levels of total sugar were seen among breakfasts, which ranged on average from 24 g (Western region) to 34 g in the Mid-Atlantic region (e.g., Delaware, District of Columbia, Maryland, New Jersey, Pennsylvania, Virginia, West Virginia). When examining urbanicity, rural school districts (both large rural and rural/small town) had on average more sodium, sugar, and fiber compared with suburban and urban school districts; meals in rural districts had on average approximately an additional 50 mg of sodium, 8 g of total sugar, and 2 g of fiber compared with urban school districts. No significant differences in calories, total fat, or saturated fat were observed by urbanicity.

Table 2 presents the percent of *daily meals* that met or were close to meeting the sodium reduction targets. Most meals met Target 1 in 2019 and 2022 for both breakfast (91.0% and 93.5%, respectively) and lunch (94.0% and 94.5%, respectively). Additionally, most lunch meals met Target 1A in 2019 and 2022 (87.3% and 88.8%, respectively). The percent of compliant breakfast meals for Target 2 was 84.3% in 2019 and 89.5% in 2022 and 70.2% for Target 3 in 2019 and 80.0% in 2022. Compliance was lower for lunch, particularly for Target 3 (9.6% in 2019 and 18.0% in 2022). In secondary analyses examining the percentage of daily meals compliant by region, the West and Mountain Plains had the highest odds of compliance for breakfast and lunch for both 2019 and 2022, while the Midwest and Southeast had lower odds of compliance (data not shown). Additionally, compliance by urbanicity was examined. All school districts, regardless of urbanicity, had over 85% compliance for Targets 1 and 1A in 2019 and 2022. However, differences became more pronounced for Targets 2 and 3, with urban school districts generally having the highest odds of compliance with Targets 2 and 3, and rural school districts having a lower odds of compliance with Targets 2 and 3.

When examining the percent of *weekly* menus that met or were close to meeting sodium reduction targets, trends were similar compared with the percent of *days* meeting (or close to meeting) the targets (Table 3). Over 90% of weekly menus met Target 1 for both breakfast and lunch in 2019 and 2022. For breakfast, Target 1 compliance in 2019 and 2022 was 94.6% and 97.1%, respectively, and, for lunch, 96.0% in 2019 and 99.1%, respectively. For Target 1A, 92.0% of weeks met the target in 2019 and 95.8% in 2022. Weekly compliance with Target 2 for breakfast was 85.8% and 94.0% in 2019 and 2022, respectively. Lunch compliance with Target 2 was slightly lower than breakfast; 69.8% of weeks met the target in 2019, and 73.7% of weeks met the target in 2022. For breakfast, 68.3% of weeks met Target 3 in 2019, and 81.2% of weeks met the target in 2022. When examining lunch, only 3.9% of weeks met Target 3 in 2019 and 12.3% of weeks met the target in 2022. In secondary analyses examining the percentage of weeks compliant by region and by urbanicity, the results were similar to the analyses examining compliance among the daily averages.

In secondary analyses, condiments were included in the estimates of the percent of average daily and weekly meals that met or were close to meeting sodium reduction targets (Supplemental Table S5). Compliance was lower for all sodium targets for both breakfast and lunch after accounting for condiments, although most meals and weeks (at least 80%) still met Targets 1 and 1A. For example, 99.2% of weekly menus complied with Target 1 for lunch in 2022, but this decreased to 97.0% after accounting for condiments (a 2.2 percentage-point decrease). Additionally, 95.8% of weekly menus were compliant with Target 1A in 2022 for lunch, but this decreased to 90.2% after accounting for condiments (a 5.8 percentage-point decrease). Similarly, compliance was lower with Targets 2 and 3 after accounting for condiments. For example, 73.7% of weekly menus complied with Target 2 for lunch in 2022, but after accounting for condiments, this decreased to 56.3% (a 23.6 percentage-point decrease). Compliance for Target 3 for lunch was also lower after incorporating condiments with 14.8% of meals (versus 18.0% before accounting for condiments, equating to a 17.8 percentage-point decrease) and 11.2% of weeks (compared to 12.3% of weeks prior to the incorporation of condiments, an 8.9 percentage-point decrease).

## 4. Discussion

This study found that, despite the exigencies of the COVID-19 pandemic and the waivers that were provided, compliance with school meal standards was largely maintained, and in some cases, modest improvements were made (for example, sodium levels decreased by 9.3% for breakfast and 4.8% for lunch between 2019 and 2022). Additionally, this study found that most weeks (and average daily meals) met sodium Targets 1 and 1A for breakfast and lunch in both 2019 and 2022, although this decreased slightly when condiments were included. In general, compliance with breakfast was higher than lunch for all targets, particularly with Targets 2 and 3. Regional differences were observed with variations by nutrients, and differences by urbanicity were also found; in particular, rural school districts served meals with higher sodium, total sugar, and fiber levels.

The 2020–2025 Dietary Guidelines for Americans (DGA) currently recommends that no more than 10% of calories come from added sugars or roughly 40 g of added sugar for a 1600 calorie/day diet [25]. Our study found that breakfast and lunch provided on average 57 g of total sugar, although some of this sugar was likely naturally occurring. However, common breakfast combinations in the present dataset were high in added sugar; for example, chocolate milk + a sugary entrée (such as a waffle) + a condiment (syrup) could provide as much as 40 g of added sugar or roughly 40% of calories from added sugar (and this meal combination was frequently available in the present study). This study’s results regarding sugar in school meals are similar to a 2021 study by Fox et al. that found that 92% of schools exceeded the DGA limits on added sugars at breakfast and nearly 70% exceeded limits at lunch [26]. Currently, there is no standard for added sugars in school meals; overall, results from both time points in 2019 and 2022 strongly suggest that there is a need for limits on added sugars included in future school meal guidelines.

When examining fiber, there were no differences between the two time points for lunch, although there was a small decrease observed at breakfast. This decrease at breakfast may have been in part due to the whole-grain waivers available to schools (whole grains are a key source of fiber in school meals). School-district breakfasts had on average 3 g of fiber, and school-district lunches had approximately 8 g of fiber. DGA recommendations for children aged 4–13 range from 17 to 25 g of fiber, suggesting that many students who eat both school breakfast and lunch are likely consuming less than half these recommended levels from school meals [25]. This further highlights the need for strong whole-grain standards for school meals.

Encouragingly, sodium levels decreased slightly between 2019 and 2022. Overall, breakfast sodium targets were met more frequently than lunch targets. Additionally, this study found that many (over 85%) of daily school-district meals met Target 1 and 1A sodium standards in both 2019 and 2022. The majority of daily meals (above 80%) also met or were close to meeting the breakfast and lunch Target 2 standards in 2022, but a lower percentage of lunches met or were close to meeting the Final Target 3 standards. Similarly, a prior study examining sodium levels in a large, urban school district in New England found that the majority of the lunches selected and consumed were already in alignment with the Target 2 standards [27]. Additionally, that study found high consumption of the lower-sodium foods, which highlights the acceptability of these types of foods among students. Importantly, in the present study there was often even greater compliance with the *weekly* sodium targets compared to *daily* meal compliance in both 2019 and 2022. The majority of weekly menus (above 90%) met or were close to meeting the breakfast and lunch Target 2 standards in 2022. School districts that served higher sodium meals one day often served lower sodium meals the rest of the week to lower that week’s sodium average, which also suggests there are many lower-sodium options available to schools.

However, despite many meals meeting sodium Targets 1, 1A, and 2 in 2022, school meals still contained substantial amounts of sodium. The DGA recommends no more than 1500 mg per day for children aged 4–8 and no more than 1800 mg of sodium per day for children aged 9–13 [25]. Thus, results from this study suggest that, on average, a school lunch meal accounts for almost half (833 mg/1800 mg, 46.3%) of a child’s daily sodium intake. Similar to the top contributors of sodium among the overall U.S. population, the foods that were the greatest contributors to sodium included processed meals (e.g., hot dogs, bacon, and deli meats), cheese (e.g., grilled cheese sandwiches), bread/rolls, pizza, and Mexican mixed dishes (e.g., tacos) [28]. Of note, condiments were a key contributor to sodium, with salad dressing often adding approximately 300–650 mg to vegetable dishes. Overall, this research suggests that the implementation of further transitional sodium targets is both necessary and feasible for schools. Offering lower-sodium condiments may be an easy, first step for schools to decrease their meals’ average sodium levels and provide meals that better align with sodium targets. Funding opportunities at the local, state, and federal levels that support investments in infrastructure, equipment, and culturally appropriate culinary training can further support school efforts to provide low-sodium and appealing school meals.

This study had several limitations. First, school-district menus were examined, which may differ slightly from what was actually served in schools, given the supply chain disruptions. However, data was collected in the spring of 2022 when many of the pandemic-related supply chain issues had been resolved. Additionally, only 128 school districts were examined, although this was a nationally representative sample and nearly 9000 meals were analyzed to support more precise estimates. While some districts were lost to follow-up (as they no longer had nutrients available online), the final sample continued to have national representation across the USDA Food and Nutrition Service Regions and by district size (and over 3000 meals were analyzed during this time period). While condiments (e.g., ketchup) were included in secondary analyses, the standardized serving sizes provided by school districts were used, which can vary in both students’ selection and portion sizes consumed, so it is possible that the average contribution of condiments to school meals may differ slightly. When examining sugar, only total sugars were examined, as most school districts did not report added sugars. Future studies should further examine added sugars in school meals. Additionally, only elementary school districts were examined, and therefore future studies should also examine middle and high schools. Lastly, this analysis gave equal weight to all menu options offered (e.g., if both pizza and a chef salad were offered as the day’s entrée options, an average was created for that day’s entrée nutrient values). However, many “healthier” items, such as salads, were also high in sodium, saturated fat, and other nutrients due to salad dressing and other toppings. Future studies should examine production records to examine if there are differences using weighted averages (similar to USDA’s methodology for assessing compliance) to account for the amount of food items offered.

This study also had several strengths. This study is the first to examine the nutrient content of school breakfast and lunch menus pre- and post-COVID-19 (with the pandemic recovery waivers) in a nationally representative sample of school districts. This study also used a pre/post design, comparing nutrients from pre-COVID-19 school-district lunch and breakfast menus (SY 2019–2020) to menus during the pandemic (SY 2021–2022). The study was further strengthened by the large number of menus analyzed and representation among school districts in all regions of the U.S. with variation by school size and urbanicity, thus enhancing the study’s generalizability. As supply chain issues continue to resolve, future studies should continue to assess school districts’ ability to provide meals that align with strong school meal standards.

## 5. Conclusions

To our knowledge, this is the first nationally representative study to examine the nutrient content of school meals and alignment with sodium targets pre- and post-pandemic (in the presence of the pandemic recovery waivers for school meals). The results of this study are encouraging; many meals were already in alignment with lower sodium targets in 2019 and are even more so in 2022. Moreover, simple strategies, such as offering lower-sodium condiments, can support efforts to further reduce sodium in school meals. Lastly, this study suggests that there is a considerable amount of sugar in school meals, especially in breakfast, which highlights that the USDA should consider limits on added sugars in school meals.

## Figures and Tables

**Table 1:** Average nutrient contents of school districts’ breakfasts and lunches in a nationally representative sample of elementary (K-5), comparing fall 2019 to spring 2022 (*n* = 91 school districts).

Nutrients				
**Calories (kcal)**				
	2019 Average (95% CI) ^1^	2022 Average (95% CI) ^1^	Average Difference (% Change)	*p*-Value ^2^
Breakfast	373 (370–376)	355 (351–358)	**−18 (−4.8%)**	**<0.0001**
Lunch	582 (577–586)	575 (571–580)	−7 (−1.2%)	0.06
**Total Fat (g)**				
Breakfast	7.2 (7.0–7.3)	7.0 (6.9–7.2)	−0.2 (−2.8%)	0.2
Lunch	16.7 (16.4–17.0)	16.9 (16.6–17.2)	+1.2 (+1.2%)	0.4
**Saturated Fat (g)**				
Breakfast	2.8 (2.7–2.9)	2.7 (2.6–2.7)	**−0.1 (−3.6%)**	**0.006**
Lunch	5.7 (5.6–5.8)	5.9 (5.8–6.0)	**+0.2 (+3.5%)**	**0.006**
**Sodium (mg)**				
Breakfast	399 (394–404)	362 (356–368)	**−37 (−9.3%)**	**<0.0001**
Lunch	875 (865–885)	833 (822–844)	**−42 (−4.8%)**	**<0.0001**
**Total Sugar (g)**				
Breakfast	30.3 (29.6–30.9)	29.2 (28.5–30.0)	**−1.1 (−3.6%)**	**0.04**
Lunch	29.2 (28.5–29.9)	27.6 (26.9–28.4)	**−1.6 (−5.5%)**	**0.003**
**Fiber (g)**				
Breakfast	3.6 (3.5–3.7)	3.4 (3.3–3.5)	**−0.2 (−5.6%)**	**0.004**
Lunch	7.5 (7.3–7.8)	7.8 (7.5–8.1)	+0.3 (+4.0%)	0.2

Note: Boldface indicates statistical significance (*p* < 0.05). ^1^ Calculated using least squares mean regression. ^2^ Calculated using analysis of variance, accounting for repeated measures within school districts, adjusting for school-district characteristics (urbanicity and region of the United States).

**Table 2:** Percent of *daily meals* that met or were close to meeting sodium reduction targets in a nationally representative sample of elementary (K-5) school-district breakfast and lunch menus in 2019 and 2022.

	SBP		NSLP	
	% of Meals That Met Target	% of Meals That Were Close to Meeting Target (within 10%)	% of Meals That Met Target	% of Meals That Were Close to Meeting Target (within 10%)
	2019 (*n* = 128 districts)			
Target 1 ^a^	91.0	93.8	94.0	97.1
Target 1A ^b^	N/A	N/A	87.3	93.5
Target 2 ^c^	84.3	90.4	65.2	77.8
Final Target 3 ^d^	70.2	81.9	9.6	18.8
	2022 (*n* = 91 districts)			
Target 1 ^a^	93.5	96.0	94.5	97.7
Target 1A ^b^	N/A	N/A	88.8	94.1
Target 2 ^c^	89.5	92.7	68.6	82.7
Final Target 3 ^d^	80.0	87.2	18.0	27.0

^a^ Target 1 requirements are ≤540 mg of sodium per breakfast meal and ≤1230 mg of sodium per lunch meal averaged over the week, currently in effect. ^b^ Target 1A requirements are ≤1110 mg of sodium per lunch meal averaged over the week beginning 1 July 2023. ^c^ Target 2 requirements are ≤485 mg of sodium per breakfast meal and ≤935 mg of sodium per lunch meal averaged over the week (note: this is no longer a target for school meals). ^d^ The final target requirements are ≤430 mg of sodium per breakfast meal and ≤640 mg of sodium per lunch meal averaged over the week (note: this is no longer a target for school meals).

**Table 3:** Percent of *weekly menus* ^a^ that met or were close to meeting sodium reduction targets in a nationally representative sample of elementary (K-5) school-district breakfast and lunch menus in 2019 and 2022.

	SBP		NSLP	
	% of Weekly Menus That Met Target	% of Weekly Menus That Were Close to Meeting Target (within 10%)	% of Weekly Menus That Met Target	% of Weekly Menus That Were Close to Meeting Target (within 10%)
	2019 (*n* = 128 districts)			
Target 1 ^b^	94.6	96.0	96.0	98.7
Target 1A ^c^	N/A	N/A	92.0	95.9
Target 2 ^d^	85.8	94.1	69.8	83.6
Final Target 3 ^e^	68.3	83.0	3.9	8.6
	2022 (*n* = 91 districts)			
Target 1 ^b^	97.1	98.2	99.2	99.8
Target 1A ^c^	N/A	N/A	95.8	99.1
Target 2 ^d^	94.0	96.9	73.7	90.5
Final Target 3 ^e^	81.2	91.4	12.3	20.7

^a^ Weekly menus were calculated based on the calendar year. ^b^ Target 1 requirements are ≤540 mg of sodium per breakfast meal and ≤1230 mg of sodium per lunch meal averaged over the week, currently in effect. ^c^ Target 1A requirements are ≤1110 mg of sodium per lunch meal averaged over the week beginning 1 July 2023. ^d^ Target 2 requirements are ≤485 mg of sodium per breakfast meal and ≤935 mg of sodium per lunch meal averaged over the week (note: this is no longer a target for school meals). ^e^ The final target requirements are ≤430 mg of sodium per breakfast meal and ≤640 mg of sodium per lunch meal averaged over the week (note: this is no longer a target for school meals).

## Data Availability

The data presented in this study are available on request from the corresponding author.

## Supplementary Materials

The following supporting information can be downloaded at: https://www.mdpi.com/article/10.3390/nu14245386/s1, Figure S1: Timeline of Sodium Standard for School Meals. Figure S2: USDA Food and Nutrition Service Region^1^. Table S1: USDA Sodium Targets for School Meals. Table S2: Average (SD) Student Population Size Among *n* = 128 School Districts in a Nationally Representative Sample by Region and Size Tier. Table S3: Elementary Schools Excluded from the 2022 Analysis (*n* = 37).* Table S4: Average nutrient contents of school breakfasts and lunch, compared to USDA standards, in a nationally representative sample of elementary (K-5) school breakfast and lunch menus in 2019 and 2022. Table S5: Percent of average daily meals and weekly meals, including condiments that met or were close to meeting sodium reduction targets in a nationally representative sample of elementary (K-5) school breakfast and lunch menus in 2022.
