# Supplementary material for: Nutrient Content and Compliance with Sodium Standards in Elementary School Meals in the United States Pre- and Post-COVID-19
Source: Nutrients. 2022 Dec 19;14(24):5386. doi: 10.3390/nu14245386 (PMC9784979; doi:10.3390/nu14245386)
Supplement: Supplementary file 1 [file nutrients-14-05386-s001.zip › nutrients-2045563-Supplementary.pdf]

Supplemental Table S1. USDA Sodium Targets for School Meals.

Age / Grade Group	Target 1 (SY 2022-23)[1] (mg)	Interim Target 1A (SY 2023-24)[2] (mg)	Target 2[3] (mg)	Target 3 (Final)[4] (mg)
**BREAKFAST**				
K-5	≤ 540	N/A	≤ 485	≤ 430
6-8	≤ 600	N/A	≤ 535	≤ 470
9-12	≤ 640	N/A	≤ 570	≤ 500
**LUNCH**				
**Age / Grade Group**	**Target 1 (SY 2022-23)[1] (mg)**	**Interim Target 1A (SY 2023-24)[2] (mg)**	**Target 2[3] (mg)**	**Target 3 (Final)[4] (mg)**
K-5	≤ 1,230	≤ 1,110	≤ 935	≤ 640
6-8	≤ 1,360	≤ 1,225	≤ 1,035	≤ 710
9-12	≤ 1,420	≤ 1,280	≤ 1,080	≤ 740

SY: School year; USDA: United States Department of Agriculture

[1]Target 1 standards were established in the 2012 final rule and implemented during SY 2014-15.

[2]Target 1A standards were established in the 2022 USDA "Transitional Standards for Milk, Whole Grains and Sodium" to be implemented during SY 2023-24 for lunch (breakfasts will remain at the original Target 1 standards during SY 2023-24).

[3]Target 2 standards were originally established in the 2012 final rule to be implemented during SY 2017-18, but were then delayed and ultimately eliminated by the USDA. (The USDA has not yet issued a proposed rule for the longer-term school meal nutrition standards.) https://www.fns.usda.gov/cn/fr-121218

[4]Target 3 standards were originally established in the 2012 final rule to be implemented during SY 2022-23, but were later eliminated by the USDA. https://www.fns.usda.gov/cn/fr-121218

Supplemental Table S2. Average (SD) Student Population Size Among n=128 School Districts in a Nationally Representative Sample by Region and Size Tier.

Size Tier	Region							
	Northeast (n=14)	Mid-Atlantic (n=20)	Southeast (n=22)	Midwest (n=16)	Mountain Plains (n=17)	Southwest (n=20)	West (n=19)	Overall (n=128)
**Tier 1 (n=14)**	164 (183)	220 (106)	284 (134)	328 (183)	288 (177)	171 (89)	311 (146)	252 (134)
**Tier 2 (n=14)**	1,225 (322)	1,360 (193)	1,322 (529)	1,168 (225)	1,072 (314)	1,092 (187)	1,700 (228)	1,277 (305)
**Tier 3 (n=14)**	2,370 (504)	2,533 (434)	2,122 (33)	2,232 (153)	2,982 (236)	2,880 (1,198)	4,005 (383)	2,732 (739)
**Tier 4 (n=14)**	5,194 (6)	5,288 (374)	4,576 (57)	5,257 (480)	5,976 (1,245)	5,853 (1,107)	4,983 (53)	5,304 (677)
**Tier 5 (n=14)**	7,574 (285)	8,194 (315)	8,462 (560)	8,382 (2,107)	7,810 (120)	8,633 (2,069)	8,559 (1,294)	8,230 (993)
**Tier 6 (n=14)**	12,894 (3,310)	14,302 (3,946)	15,696 (1,198)	11,468 (959)	14,011 (4,675)	17,076 (2,039)	11,648 (232)	13,871 (2,854)
**Tier 7 (n=13)**	24,439†ƚ	24,452 (1,178)	23,529 (3,692)	24,026 (2,848)	21,766 (2,830)	23,804 (2,848)	24,540 (2,614)	23,898 (2,263)
**Tier 8 (n=8)**	*	35,730 (6,812)	30,312 (2,587)	34,284†	33,661 (5,853)	32,370 (703)	32,496 (6,476)	33,038 (4,037)
**Tier 9 (n=5)**	*	56,942 (6,087)	46,464 (722)	*	43,328†	46,252 (5,067)	61,584†	50,528 (7,456)
**Tier 10 (n=5)**	*	75,799 (10,996)	71,402 (10,827)	*	*	75,221†	*	73,924 (8,056)
**Tier 11 (n=7)**	470,656†	*	140,734 (25,132)	169,384†	*	105,649†	228,826 (112,607)	212,115 (131,697)
**Overall (n=128)**	39,567 (124,259)	22,582 (25,612)	31,355 (41,920)	19,337 (41,250)	12,851 (13,591)	22,857 (27,856)	36,608 (74,388)	26,374 (56,284)

† *Only one district met the sample selection criteria.*
ƚ *This district operates two distinct food service models: a "scratch cooked" menu and a "pre-packaged" menu. Both menus were included as "separate" districts.*
* *No district met the sample selection criteria.*

Supplemental Table S3. Elementary Schools Excluded from the 2022 Analysis (n=37).*

2022 EXCLUDED SCHOOLS		
**School District**	**Region**	**Tier Size**
School 1	Mid-Atlantic Region	6
School 2	Mid-Atlantic Region	10
School 3	Mountain Plains Region	5
School 4	Mountain Plains Region	7
School 5	Midwest Region	3
School 6	Midwest Region	3
School 7	Midwest Region	4
School 8	Midwest Region	6
School 9	Midwest Region	7
School 10	Midwest Region	8
School 11	Midwest Region	11
School 12	Northeast Region	1
School 13	Northeast Region	2
School 14	Northeast Region	3
School 15	Northeast Region	4
School 16	Northeast Region	6
School 17	Southeast Region	1
School 18	Southeast Region	1
School 19	Southeast Region	2
School 20	Southeast Region	3
School 21	Southeast Region	4
School 22	Southeast Region	4
School 23	Southeast Region	5
School 24	Southeast Region	6
School 25	Southeast Region	8
School 26	Southeast Region	9
School 27	Southwest Region	2
School 28	Southwest Region	2
School 29	Southwest Region	4
School 30	Southwest Region	4
School 31	Southwest Region	7
School 32	Southwest Region	8
School 33	Southwest Region	9

School 34	West Region	1
School 35	West Region	2
School 36	West Region	4
School 37	West Region	9

*Schools were excluded because menu and/or nutrient data were not found for 2022.

Supplemental Table S4. Average nutrient contents of school breakfasts and lunch, compared to USDA standards, in a nationally representative sample of elementary (K-5) school breakfast and lunch menus in 2019 and 2022.

Meal Type	Year	USDA Standards Versus Nutrient Averages	Nutrients[a]					
			Calories (range [kcal])	Saturated Fat[b] (g)	Sodium Target 1 (mg)	Sodium Target 1A (mg)[c]	Sodium Target 2 (mg)[d]	Sodium Target 3 (mg)[d]
Breakfast	2019	USDA Standard	350 - 500	3.9 - 5.6	≤ 540	N/A	≤ 485	≤ 430
		Average (95% CI)	373 (370 – 376)	2.8 (2.7 – 2.9)	399 (394 – 404)			
		Difference (Average - USDA Standard)	-127[e] – +23[f]	-2.8 – -1.1	-141	N/A	-86	-31
		USDA Breakfast Standards	Met Requirement	Met Requirement	Met Requirement	N/A	Met Target[d]	Met Target[d]
	2022	USDA Standard	350 - 500	3.9 - 5.6	≤ 540	N/A	≤ 485	≤ 430
		Average (95% CI)	355 (351 – 358)	2.7 (2.6 – 2.7)	362 (356 – 368)			
		Difference (Average - USDA Standard)	-145 – +5	-2.9 – -1.2	-178	N/A	-123	-68
		USDA Breakfast Standards	Met Requirement	Met Requirement	Met Requirement	N/A	Met Target[d]	Met Target[d]
Lunch	2019	USDA Standard	550 - 650	6.1 - 7.2	≤ 1,230	≤ 1,110	≤ 935	≤ 640
		Average (95% CI)	582 (577 – 586)	5.7 (5.6 – 5.8)	875 (865 – 885)			
		Difference (Average - USDA Standard)	-68 – +32	-1.5 – -0.4	-355	-235	-60	235
		USDA Lunch Standards	Met Requirement	Met Requirement	Met Requirement	Met Target[c]	Met Target[d]	Did not Meet Target[d]
	2022	USDA Standard	550 - 650	6.1 - 7.2	≤ 1,230	≤ 1,110	≤ 935	≤ 640

		Average (95% CI)	575<br>(571 – 580)	5.9<br>(5.8 – 6.0)	833<br>(822 – 844)			
		Difference<br>(Average - USDA Standard)	-75 – +25	-1.3 – -0.2	-397	-277	-102	193
		USDA Lunch Standards	Met Requirement	Met Requirement	Met Requirement	Met Target[c]	Met Target[d]	Did not Meet Target[d]

[a]There are no USDA nutrient requirements for total fat, fiber, or total sugar for reimbursable breakfast and lunch meals. Nutrient targets are averaged over the week.

[b]Based on saturated fat limits of <10% of the average weekly meal maximum for calories (350-500 kcal for breakfast and 550-650 kal for lunch for grades K-5).

[c]This target will go into effect in SY 2023-24 for lunch (breakfasts will remain at the original Target 1 standards during this time).

[d]This is no longer a USDA target for school meals.

[e]Calculated based on the upper range for the USDA requirement.

[f]Calculated based on the lower range for the USDA requirement.

Supplemental Table S5. Percent of average *daily meals* and *weekly meals,* including condiments that met or were close to meeting sodium reduction targets in a nationally representative sample of elementary (K-5) school breakfast and lunch menus in 2022.

	SBP		NSLP	
**Target**	**% that Met Target**	**% that were Close to Meeting Target (within 10%)**	**% that Met Target**	**% that were Close to Meeting Target (within 10%)**
**Average Daily Meals**				
**Target 1**[a]	93.4	95.9	89.9	95.4
**Target 1a**[b]	N/A	N/A	82.3	89.3
**Target 2**[c]	88.4	92.6	55.7	72.0
**Final Target 3**[d]	77.6	86.0	14.8	20.5
	**Average Week**			
**Target 1**[a]	97.1	98.2	97.0	98.8
**Target 1a**[b]	N/A	N/A	90.2	96.7
**Target 2**[c]	93.2	96.6	56.3	77.0
**Final Target 3**[d]	78.8	90.3	11.2	16.1

[a]Target 1 requirements are ≤540 mg of sodium per breakfast meal and ≤1,230 mg of sodium per lunch meal averaged over the week.

[b]Target 1a requirements are ≤ 1,110 mg of sodium per lunch meal averaged over the week.

[c]Target 2 requirements are ≤ 485 mg of sodium per breakfast meal and ≤ 935 mg of sodium per lunch meal averaged over the week (note: this is no longer a target for school meals).

[d]The final target requirements are ≤ 430 mg of sodium per breakfast meal and ≤ 640 mg of sodium per lunch meal averaged over the week (note: this is no longer a target for school meals).

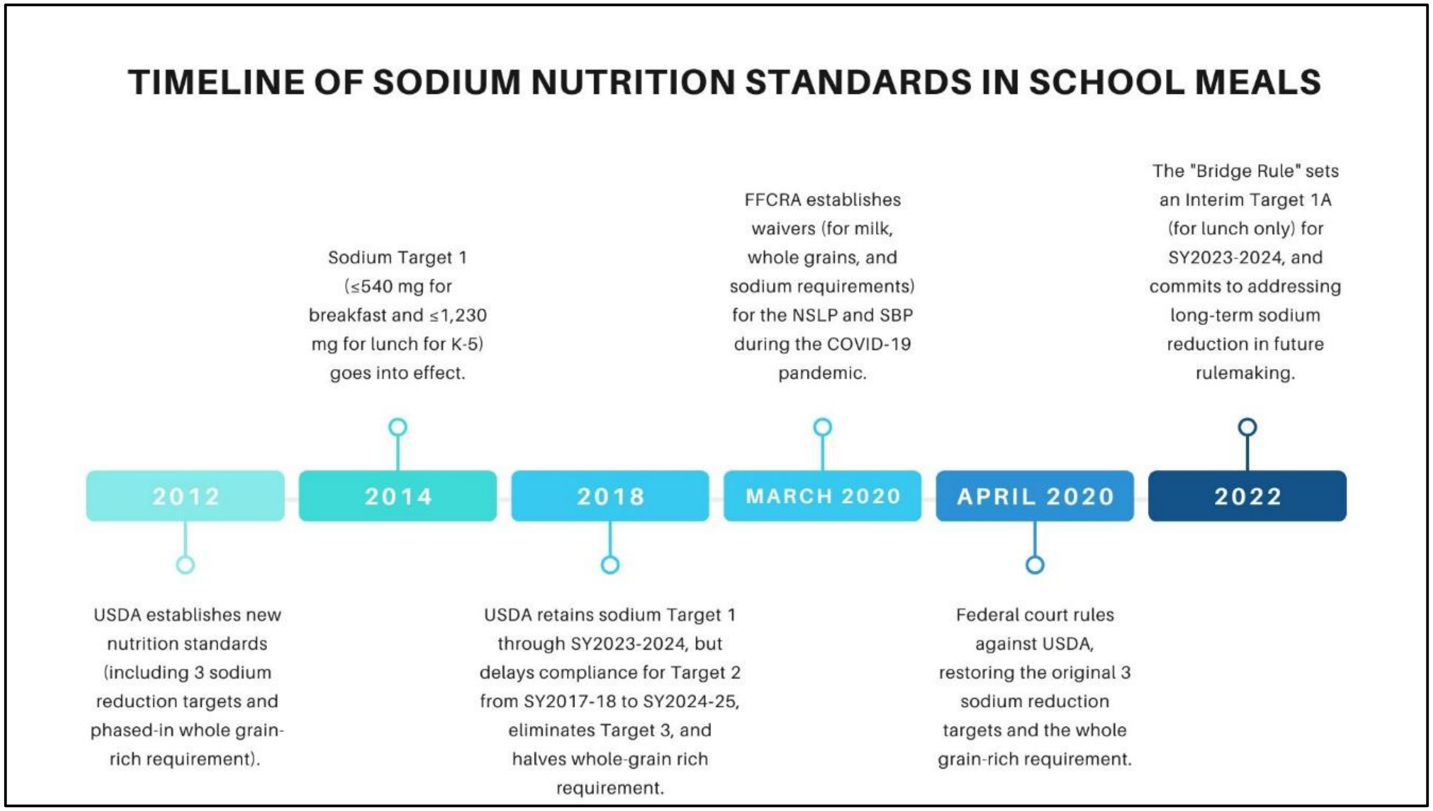

Supplemental Figure S1. Timeline of Sodium Standard for School Meals.

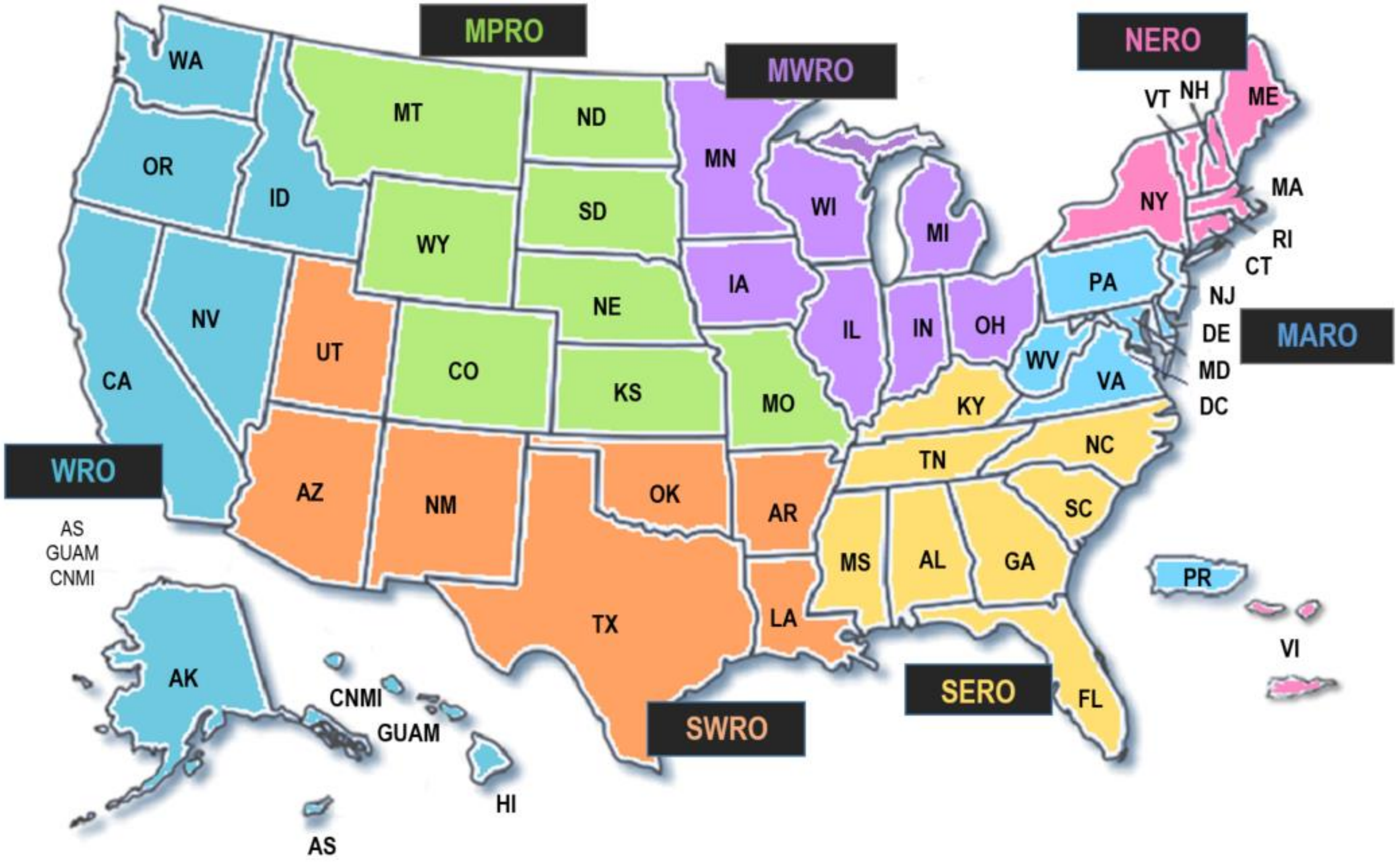

Supplemental Figure S2. USDA Food and Nutrition Service Region[1]

[1] Source: https://www.fns.usda.gov/fns-regional-offices
